# Thrombotic microangiopathy associated with Valproic acid toxicity

**DOI:** 10.1186/s12882-017-0677-4

**Published:** 2017-08-03

**Authors:** Sean A. Hebert, Timothy P. Bohan, Christian L. Erikson, Rita D. Swinford

**Affiliations:** 1 Department of Internal Medicine and Pediatrics, Division of Nephrology, McGovern Medical School at The University of Texas Health Science Center at Houston (UTHealth), Children’s Memorial Hermann Hospital, 6431 Fannin Street, MSB 3.121, Houston, TX USA; 2 Pediatric Neurology, Memorial Hermann Texas Medical Center, Children’s Memorial Hermann Hospital, 6400 Fannin Street, Ste 2740, Houston, TX USA; 3 Department of Pediatrics, Division of Critical Care Medicine, Texas Children’s Hospital, The Woodlands 17600 I-45 South, WL 640, Conroe, TX USA; 4 Department of Pediatrics, Division of Nephrology, McGovern Medical School at The University of Texas Health Science Center at Houston (UTHealth), Children’s Memorial Hermann Hospital, 6431 Fannin Street, MSB 3. 121, Houston, TX USA

**Keywords:** Thrombotic microangiopathy, Drug-induced thrombotic Microangiopathy, Valproic acid toxicity, Case report

## Abstract

**Background:**

Thrombotic microangiopathy (TMA) is a serious, sometimes life-threatening disorder marked by the presence of endothelial injury and microvascular thrombi. Drug-induced thrombotic microangiopathy (DI-TMA) is one specific TMA syndrome that occurs following drug exposure via drug-dependent antibodies or direct tissue toxicity. Common examples include calcineurin inhibitors Tacrolimus and Cyclosporine and antineoplastics Gemcitabine and Mitomycin. Valproic acid has not been implicated in DI-TMA. We present the first case of a patient meeting clinical criteria for DI-TMA following admission for valproic acid toxicity.

**Case presentation:**

An adolescent male with difficult to control epilepsy was admitted for impaired hepatic function while on valproic acid therapy. On the third hospital day, he developed severe metabolic lactic acidosis and multiorgan failure, prompting transfer to the pediatric intensive care unit. Progressive anemia and thrombocytopenia instigated an evaluation for thrombotic microangiopathy, where confirmed by concomitant hemolysis, elevated lactate dehydrogenase (LDH), low haptoglobin, and concurrent oliguric acute kidney injury. Thrombotic thrombocytopenic purpura was less likely with adequate ADAMTS13. Discontinuing valproic acid reversed the anemia, thrombocytopenia, and normalized the LDH and haptoglobin, supporting a drug-induced cause for the TMA.

***Conclusion*:**

To the best of our knowledge, this is the first report of drug-induced TMA from valproic acid toxicity.

## Background

The diagnosis of drug-induced thrombotic microangiopathy (DI-TMA) is often complex and difficult. TMAs are a group of heterogeneous disorders defined not only by the presence of microvascular thrombi, thrombocytopenia and hemolysis involving predominantly the kidney but all organ systems [1, 2]. Considered a prothrombotic condition secondary to an underlying disturbance of coagulation and complement systems, etiologies include: thrombotic thrombocytopenic purpura (TTP), a systemic disorder of microvascular thrombosis of ADAMTS13 deficiency, hemolytic uremic syndrome (HUS), with and without complement mutations, characterized by nonimmune hemolytic anemia, thrombocytopenia, and renal failure, and drug induced TMA (DI-TMA), through either a direct toxic effect or duration dependent toxicity. [3]

Valproic acid (VPA), an anticonvulsive agent for epileptic syndromes, has adverse consequences associated with its use; most mild and transient but others serious, affecting embryonic development and still others, uncommonly, causing significant disorders of hepatic and pancreatic dysfunction, Fanconi syndrome and weight gain [4]. Hematological disorders are seen in children treated with VPA, including thrombocytopenia, platelet dysfunction, Von Willebrand disease, Factor XIII deficiency, hypofibrinogenemia and vitamin K-dependent factor deficiency. A dual etiology is responsible: direct toxicity of VPA on bone marrow and VPA’s inclusion with and modification of the platelet membrane [4].

Acute TMA in conjunction with VPA toxicity has, to our knowledge, not been reported. We propose that VPA’s ability to modify cellular membranes may explain the TMA and acute kidney injury seen in our patient.

## Case presentation

A 16 year old male with epilepsy and variable response to anti-convulsants presented to our hospital. Previously controlled with a combination of valproic acid (VPA) and levetiracetam for many years, his seizures recurred despite high therapeutic levels, prompting dose modifications. At a routine clinic appointment, he complained of severe emesis, prompting admission. There his VPA level was low, 46 μg/ml (normal range 50–100 μg/ml). He was bolused with VPA (15 mg/kg), achieving a toxic VPA level of 137 μg/ml, remaining elevated (130 μg/ml) for 16 h. The typical half-life for VPA is 9–16 h in adolescents. His ALT (234 unit/L) and AST (63 unit/L) were mildly elevated so intravenous (IV) L-carnitine was initiated for VPA-induced hepatotoxicity.

Subsequently an abrupt deterioration manifested, a severe anion gap metabolic acidosis (pH 7.05, pCO2 24 mmHg, serum bicarbonate 12 mEq/L, serum lactic acid 16.9 mMol/L), and acute kidney injury (creatinine increased from 1.2 mg/dL on admission to 2.4 mg/dL). Now in the pediatric intensive care unit, despite adequate volume resuscitation and correction of pH, his creatinine rose to 2.6 mg/dL.

Laboratory findings concomitantly consistent with a TMA: acute kidney injury, anemia (hemoglobin 9.1 to 8.1 g/dL), thrombocytopenia (platelets 25,000 K/CMM), an elevated LDH (350 unit/L) (normal range 98–192 unit/L) and suppressed haptoglobin (<8 mg/dL) with reticulocyte count of 6.5%, reflecting adequate bone marrow response, all supported a diagnosis of acute hemolytic anemia. Peripheral blood smear showed mild macrocytic anemia, mild anisopoikilocytosis with target cells and rare schistocytes, platelets were markedly decreased. ADAMTS 13 activity reported 59% enzyme function (normal >67%). Complement levels, low C3 55 mg/dL (normal 88–201) and normal C4 30 mg/dL, suggested alternative pathway activation.

**Fig. 1:**
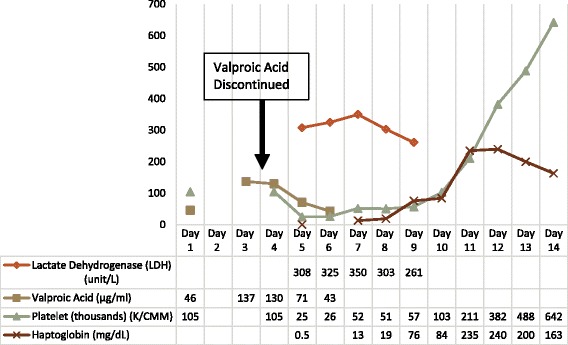
Laboratory values during hospitalization

Following discontinuation of VPA, levels normalized in five days. Concurrently, his serum creatinine, serum bicarbonate, hemoglobin and haptoglobin improved to normal ranges (Fig. 1). Complement evaluation was entertained, but ultimately not pursued when C3 normalized to 159 mg/dL after medication discontinuation. His anti-epileptic regimen was changed to Vimpat (lincosamide) and Keppra (levetiracetam), and he remained free of TMA.

## Discussion

Valproic acid is an effective anticonvulsive agent with several adverse consequences. Thrombocytopenia is the most common hematologic adverse effect, with incidence varying from 5% to 60% [5]. In 2007, Beydoun and Nasreddine illustrated the association between VPA toxicity and thrombocytopenia. The probability of developing thrombocytopenia increases when trough VPA levels are above 130 μg/ml [6]. While our patient’s prolonged VPA level elevation above 130 μg/ml are consistent with these studies, the microangiopathic hemolytic anemia, anuria, and acute kidney injury reflect a more severe hematologic etiology, consistent with thrombotic microangiopathy (TMA) syndrome.

TMA syndromes are extraordinarily diverse, divided into hereditary and acquired etiologies. Universally, TMA syndromes are incited by microvascular endothelial injury leading to arteriolar and capillary thrombosis and subsequent organ injury. DI-TMA occurs via 2 mechanisms, either non-dose related immunologic reactions or toxic-dose related reactions. While toxic-dose mediated TMA is gradual in onset, renal failure can be seen over weeks to months, immune-reaction TMA is more sudden [7].

Drug-mediated immune reactions leading to TMA were first recognized in 1980 after documented exposures to quinine [7]. Quinine antibodies included immunoglobulin G (IgG) or IgM, reactive with either glycoprotein Ib/IX or IIb/IIIa or both [8, 9]. Antibodies against granulocytes, lymphocytes, and endothelial cells have been described [9].

VPA, a branched-chain carboxylic acid, is structurally and chemically similar to fatty acid constituents of cell membranes. Sandler et al. proposed this similarity would lead to production of immunoglobulin M (IgM) antibodies against circulating thrombocytes with decreasing antibody titers after recovery [5]. For many years, Sandler’s proposal remained hypothetical. Barr et al. affirmed Sandler’s proposal when he documented increased levels of IgG platelet antibodies in half of patients receiving VPA, reflecting increased platelet destruction [10].

VPA is also shown to affect the mean corpuscular volume (MCV) of erythrocytes. Konig et al. studied 3 anticonvulsant therapies (valproic acid, carbamazepine, or phenobarbital) effect on erythrocyte cell membranes. VPA was the only anti-epileptic drug (AED) to alter the fatty acid composition of erythrocytes [11]. Affecting membrane fluidity and altering receptor proteins expressed on both the inner and outer surfaces of the erythrocyte cell membrane provides the nidus for immunoglobulin directed destruction of erythrocytes.

We performed a literature review searching for prior documentation of TMA-associated with VPA therapy. Results revealed 4 papers, three reporting isolated thrombocytopenia cases with VPA toxicity as previously mentioned. The fourth highlighted TTP and anemia in a breast fed healthy male infant whose mother was treated with VPA, unique for two reasons. First, a simultaneous decrease in both platelets and hemoglobin, not typical of either thrombocytopenia from VPA or idiopathic thrombocytopenic purpura (ITP). Secondly, no concentration-dependent toxic effect was seen since the infant’s serum VPA concentration remained low during the entire clinical course. Similar to our patient, thrombocytopenia and anemia resolved with removal of the drug, in this case cessation of breastfeeding. [12] After thorough medication review, an undetermined etiology makes VPA or its metabolites a possible culprit.

First recognized in 1979, there is increasing awareness of pancreatitis with disseminated intravascular coagulation (DIC) [13]. By July 2010, the FDA introduced new black box warnings for VPA, pancreatitis, however, the diagnosis of pancreatitis requires strict criteria. Explicitly, amylase and/or lipase elevation should be greater than 3 times the upper limit of normal, imaging studies should show evidence of inflammation, and, if performed, pancreatic damage should be visualized during surgery [14]. While still possible, our patient’s lab values and absent inflammation on imaging, make pancreatitis unlikely. Using the International Society for Thrombosis and Hemostasis DIC scoring system, our patient never met criteria for overt DIC [15].

Presenting with nausea/vomiting in up to 50% of patients, VPA hepatotoxicity occurs due to disruption of mitochondrial processes, proposed as drug-induced carnitine deficiency. As carnitine facilitates fatty acyl group transport into and out of the mitochondria, when acyl groups accumulate (as in drug intoxications), carnitine assists in transporting these acyl groups out of the mitochondria and eventually into the urine, inducing a secondary carnitine deficiency [16].

While rare and often fatal, timely initiation of intravenous L-carnitine treatment can often be life-saving. The patient’s neurologist began IV L-carnitine (50 mg/kg loading dose followed by 50 mg/kg over the next 24 h and then 100 mg/kg/day in divided doses for 7 days) within five days of admission, a favorable factor for improving hepatic function and VPA metabolism. Carnitine deficiency explains the impaired beta-oxidation of VPA allowing for immune-mediated TMA.

The presence of acute liver injury should not detract from suspicion for TMA. A subset of patients with endothelial damage have TAMOF, thrombocytopenia-associated multi-organ failure. This entity is increasingly identified in critically ill pediatric patients with signs consistent with TTP/HUS, but without significant hemolysis as seen by rare schistocytes, normal haptoglobin, and mildly suppressed ADAMTS13 activity. TAMOF exists within a spectrum of TMA syndromes, commonly defined by systemic endothelial cell destruction. Resolution of multiple organ dysfunction requires elimination of the source of inflammation [17, 18]. Swift recognition and VPA discontinuation likely saved this patient from escalation to plasma exchange and/or hemodialysis.

Further supporting our DI-TMA hypothesis, the Naranjo scale characterizes the likelihood of a true adverse drug reaction (ADR) as doubtful, possible, probable, and definite [19]. A score of 7 makes VPA a probable culprit, limited by lack of placebo and no prior conclusive reports. Secondary TMA from VPA toxicity associated with multiple organ failure is the consensus diagnosis. Our case features the first reported case of DI-TMA with VPA toxicity. We suggest increasing awareness of VPA as a TMA culprit will assist in identifying future cases.

## Conclusion

DI-TMA may be a serious complication, previously unreported, of VPA therapy and therefore important to recognize. A prerequisite of successful treatment is the correct diagnosis which in this case was defined by exclusion. With ongoing TMA and AKI, despite drug cessation, consideration for a renal biopsy and anti-complement therapy should also be given. The possibility of adverse hematologic events secondary to VPA therapy should also be kept in mind.

## Abbreviations

DI-TMA: Drug induced thrombotic microangiopathy
HAP: haptoglobin.
LDH: Lactate Dehydrogenase
TMA: thrombotic microangiopathy
VPA: valproic acid
